# Failure and recovery in dynamical networks

**DOI:** 10.1038/srep41729

**Published:** 2017-02-03

**Authors:** L. Böttcher, M. Luković, J. Nagler, S. Havlin, H. J. Herrmann

**Affiliations:** 1 ETH Zurich, Wolfgang-Pauli-Strasse 27, CH-8093 Zurich, Switzerland; 2 Center for Polymer Studies and Department of Physics, Boston University, Boston, Massachusetts 02215, USA; 3 Department of Physics, Bar-Ilan University, 52900 Ramat-Gan, Israel; 4 Departamento de Física, Universidade Federal do Ceará, 60451-970 Fortaleza, Ceará, Brazil

## Abstract

Failure, damage spread and recovery crucially underlie many spatially embedded networked systems ranging from transportation structures to the human body. Here we study the interplay between spontaneous damage, induced failure and recovery in both embedded and non-embedded networks. In our model the network’s components follow three realistic processes that capture these features: (i) spontaneous failure of a component independent of the neighborhood (internal failure), (ii) failure induced by failed neighboring nodes (external failure) and (iii) spontaneous recovery of a component. We identify a metastable domain in the global network phase diagram spanned by the model’s control parameters where dramatic hysteresis effects and random switching between two coexisting states are observed. This dynamics depends on the characteristic link length of the embedded system. For the Euclidean lattice in particular, hysteresis and switching only occur in an extremely narrow region of the parameter space compared to random networks. We develop a unifying theory which links the dynamics of our model to contact processes. Our unifying framework may help to better understand controllability in spatially embedded and random networks where spontaneous recovery of components can mitigate spontaneous failure and damage spread in dynamical networks.

Failure, damage spread and recovery crucially underlie many spatially embedded networked systems ranging from transportation structures to the human body^1^,^2^,^3^,^4^. Advances in the study of networks have led to important progress in understanding resilience and controllability in terms of the interaction between topology and various underlying spreading dynamics^5^,^6^,^7^,^8^,^9^,^10^. In the case of a simple contagion or contact process such as an epidemic, it is possible for the disease to spread from a single infected source to other neighboring individuals. On the other hand, many phenomena such as the diffusion of innovations^11^,^12^, political mobilization^13^, viral marketing^14^ and coordination games^15^ are characterized by a complex contagion where nodes need to be connected to multiple sources in order to induce a change of their state^16^,^17^. In addition to this induced transition, individuals may spontaneously change their opinion or banks can spontaneously fail^18^,^19^.

The consequences of the interplay between spontaneous damage, induced failure and recovery of components in spatially embedded systems are crucial for systemic risk^20^, controllability but have not yet been systematically explored. Many real-world networks such as power grids, computer networks and social networks are embedded in Euclidean space^4^. We here show how the process of embedding and the related characteristic link length impact the dynamics of failure-recovery processes in networks.

Our model is based on three fundamental processes (i) spontaneous failure independent of the neighborhood (internal failure), (ii) failure induced by failed neighboring nodes if their number exceeds a threshold (external failure) and (iii) spontaneous recovery (see Fig. 1). The interplay between these three processes results in a phase diagram with a metastable regime where hysteresis and switching between two coexisting states have been observed^21^. In technological systems, hysteresis effects might be potentially harmful since slight changes of the system’s control parameters can entail drastic and abrupt transitions from a seemingly globally stable state to macroscopic inactivity or large-scale outage^9^,^22^,^23^,^24^,^25^,^26^,^27^,^28^,^29^,^30^,^31^,^32^,^33^,^34^,^35^. Hysteresis and spontaneous switching between coexisting states in multistable dynamical systems has received great attention for processes ranging from decision making^36^, multistable perception^37^,^38^,^39^ over fluid phase transitions^40^, protein folding and unfolding^41^ to chemical oscillations^42^, magnetic systems^43^ and human sleep stages^44^. We therefore propose that the extent of the metastable regime in the parameter space of the phase diagram can be regarded as a measure of the system’s inherent instability. Based on this measure we find that metastability is enhanced by the degree of randomness of the underlying network. In particular, for the Euclidean lattice which is perfectly regular, hysteresis only occurs in a very small range of the spontaneous failure rate - compared to random networks with the same average degree.

Our analytical approach is based on mapping the dynamics to a generalized contact process where a certain minimum number of failed neighboring nodes is necessary to activate the induced failure^45^. This strongly suggests that the dynamics does not belong to the Ising universality class as conjectured earlier^21^. In addition, we show that our model system is inherently linked to complex contagion phenomena^16^,^17^ and cusp catastrophes^46^,^47^,^48^. Our unifying framework for random and partially embedded networks helps to better understand the dynamics in systems where spontaneous recovery can mitigate spontaneous failure and damage spread.

## Materials and Methods

**Figure 1:**
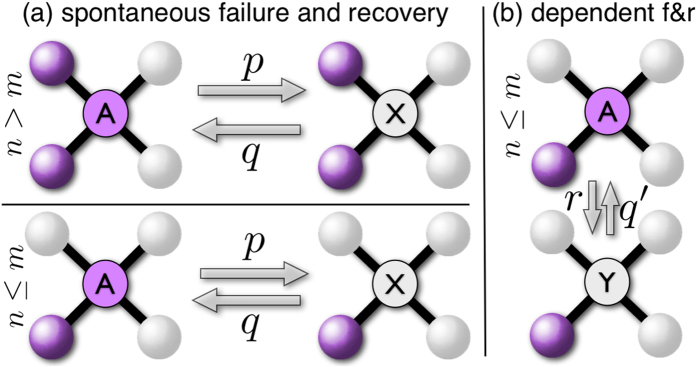
Model. (**a**) Independent of the number of *active* nodes *n*, i.e. for *n* ≤ *m* and *n* > *m*, spontaneous failure (*A* → *X*) and spontaneous recovery (*X* → *A*) takes place with rates *p* and *q*, respectively. (**b**) A node may also fail (become inactive) dependent on its neighborhood, if too few active nodes *n* ≤ *m* sustain the node’s activity (*A* → *Y* with rate *r*). In addition, a failed node *Y* recovers (*Y* → *A*) with rate *q*′. Illustration for *m* = 1. Active nodes (*A*) are purple while failed ones (*X* and *Y*) are grey.

We study a modified version of the failure-recovery model proposed in ref. 21. The failure dynamics in ref. 21 is based on two failure processes that occur with rates *r* and *p*, respectively, whereas recovery occurs after *fixed* recovery times *τ* ≠ 0 and *τ*′ = 1. We consider instead a fully rate based sequential kinetic Monte Carlo model (Gillespie algorithm)^49^,^50^ where all processes are defined by rates as follows: The system’s components (i.e. nodes) are regarded as either active (not damaged) or inactive (failed). The dynamics is based on three fundamental processes: (i) a node spontaneously fails in a time interval *dt* with probability *p*d*t* (internal failure), (ii) if fewer than or equal to *m* nearest neighbors of a certain node are active, this node fails due to external causes with probability *r*d*t* (external failure) and (iii) spontaneous recovery with probability *q*d*t* (internal recovery) or probability *q*′d*t* (external recovery). The threshold *m*, similar to threshold rules in complex contagion models^16^,^51^,^52^ determines if the neighborhood is critically damaged or healthy (Fig. 1). A low value of *m* describes the case where a large number of infected neighbors is required in order to sustain the spread of an innovation, opinion or damage. Hence, unlike in an epidemic, where a single infected neighbor can infect a susceptible node, in complex contagion processes spread requires more than one infected neighbor. Let *a*(*t*) ∈ [0, 1] denote the total fraction of *failed* nodes and *z*(*t*) = 1 − *a*(*t*) the fraction of *active* ones. Thus, *a*(*t*) = *u*_int_(*t*) + *u*_ext_(*t*) with *u*_int_(*t*) and *u*_ext_(*t*) being the fractions of internal and external failure respectively. The total fraction of failed nodes in the stationary state is referred to as *a*_*st*_. For the derivation of the mean-field rate equations we assume perfect mixing and first concentrate on the internal failure dynamics. The rate equation of internally failed nodes is given by:




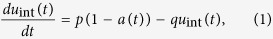




where the first term accounts for the fact that active nodes internally fail with rate *p* and the second term corresponds to the recovery of internally failed nodes.

A node is said to be located in a critically damaged neighborhood (CDN) if its number of active neighbors is smaller than or equal to *m*. External failure is only acting on nodes in a CDN. According to ref. 20, the probability that a node of degree *k* is located in a CDN is 
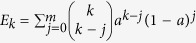
. Consequently, the time evolution of the external failure is described by:




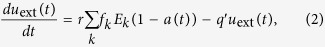




with *f*_*k*_ being the degree distribution. The first term describes the failure of active nodes in a CDN with rate *r* and the second term accounts for recovery of externally failed nodes with rate *q*′.

## Results

### Time evolution and phase-switching in embedded systems

**Figure 2:**
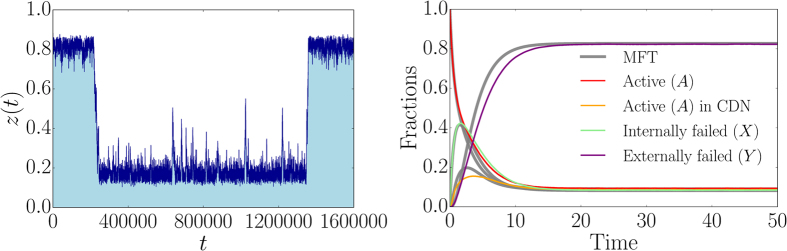
Model dynamics on a square lattice. (left) Phase switching for *p* = 0.1065, *r* = 0.95, *q* = 1.0, *q*′ = 0.1 and *m* = 1 on a square lattice with *N* = 50 × 50 nodes (*z* the fraction of active nodes). (right) Time evolution of different model compartments, i.e. nodes in a certain state (see Fig. 1), with *p* = 0.9, *r* = 0.95, *q* = 1.0, *q*′ = 0.1 and *m* = 1 for a square lattice with *N* = 1024 × 1024 nodes. All nodes are initially active, *z*(0) = 1.

The coupled mean-field rate equations Eqs (1) and (2) determine the time evolution of the dynamics as shown in Fig. 2. Internal failures first dominate the dynamics but after some time externally induced failures start becoming prevalent in the system. Interestingly, due to the system wide spread of the total failure after a transient phase, indicated by the small fraction of active nodes, the relative abundance of the nodes susceptible to external (active in a CDN) and to internal failure (active) saturate at the same level. However, the process with the higher spreading rate (here external failure with *r*/*q*′ > *p*/*q*) soon dominates the dynamics. This explains the relatively small contribution of internal failure in this parameter range which could not be observed employing the mean-field theory of ref. 21 which assumes that internal and external failure are effectively decoupled processes (case 1 ≈ *τ*′ ≪ *τ*). Therefore, our dynamical theory allows to analytically describe the time evolution of the model’s compartments, i.e. nodes in a certain state.

**Figure 3:**
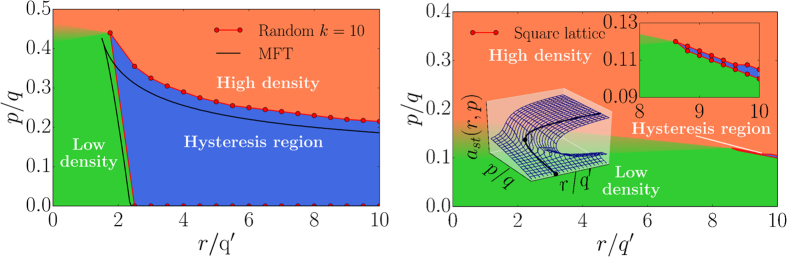
Phase diagrams of a regular random graph with *k* = 10 and a square lattice. (left) The phase space of a regular random graph with *N* = 100,000 nodes and *k* = 10, *m* = 4 (red dots) compared to the mean-field prediction (black lines). (right) The phase diagram of the square lattice (red spinodals) for *m* = 1 obtained through simulations on a system with *N* = 2048 × 2048 nodes. Inset shows a blow-up of the hysteresis region. As an inset in the lower left corner, we show a typical cusp catastrophe surface^47^ (mean-field *k* = 4, *m* = 1) whose bifurcation lines (black) enclose the hysteresis region. For both plots the recovery rates are kept constant (*q* = 1.0, *q*′ = 0.1 – no oscillatory regime since *q* > *q*′) and *r, p* are varied. Low and high density correspond to the situation where the fraction of failed nodes of the stationary state is small or large respectively. States in between are indicated by the color gradient. Note that here and in subsequent phase-space plots we plot the effective rates *p*/*q* against *r*/*q*′ which allows for a better comparison of the phase-spaces for different parameter sets but does not represent a parameter space.

In Fig. 3 we show the phase diagrams of a regular random graph with *k* = 10, *m* = 4 and of a square lattice with *k* = 4, *m* = 1. The dynamics exhibits a metastable domain (purple) and a region where the number of failed nodes only makes up a small fraction of the total number of nodes (*low density*) or where most of the nodes in the system failed (*high density*). Continuous transitions between low and high density states outside the hysteresis region are indicated by a color gradient. There exists one stable stationary state outside the hysteresis region and two stable states and one unstable state in the interior of this region as also discussed in ref. 21 and in Appendix A. The inset of Fig. 3 (right) also illustrates this fact: In the three-dimensional phase diagram one can easily identify three stationary states inside the metastable domain, enclosed by black bifurcation lines. Another observation is that the dynamics shows only one stationary state outside this region. Strictly speaking, the notion of stability and metastability is only meaningful in the thermodynamic limit. Due to fluctuations in small systems the metastable domain would look more blurry. If fluctuations are strong enough inside or close to this region, the order parameter will show switching between the two, in the thermodynamic limit, stable states independent of the initial condition (fraction of failed nodes). The situation is different in large systems with negligible fluctuations. Here, the initial condition (fraction of failed nodes) determines the final state as follows: If the initial fraction of failed nodes is larger than the fraction determining the unstable state, the dynamics will reach the state with a high density of failed nodes. If, however, the fraction of failed nodes is smaller than the one of the unstable state, the final state will be the one with a low density of failed nodes. As a consequence, inside the metastable domain, a random initial condition in a large system with negligible fluctuations will lead to a convergence towards the low density or high density failure phase. For a small system one will observe switching. For the Euclidean (square) lattice we observe phase-switching as shown in Fig. 2 (left). The fraction of active nodes *z*(*t*) undergoes rapid transitions between a phase of high and low activity. Hysteresis only occurs for large node-to-node spreading rates *r* and a very narrow range of spontaneous failures rates *p*. Inside this metastable domain rapid and unpredictable phase-switching occurs. In addition, crossing this region results in abrupt and dramatic transitions and it might not be possible to go back to the previous state following the same path. As an example, one can consider a nearly healthy population with a varying spontaneous infection rate which can cause the population to undergo a catastrophic transition to a highly infected state by crossing into the hysteresis region. Going back to the healthy state might not be as easy as just retracing the path followed before. The smaller the hysteresis region the less likely it is for the system to end up in this scenario. Dynamics in the hysteresis region are manifested in two ways: (1) In finite systems, random phase switching between two unstable states is observed where the mean of the random switching times increases exponentially with system size^21^. (2) In the thermodynamic limit no switching is observed but the initial configuration and small random events in the initial temporal evolution of the system determine to which of the two stationary states the system converges. The latter behavior is characteristic for non-self-averaging spin glasses^53^,^54^,^55^. As an inset in Fig. 3 (right), we show the relation to cusp catastrophe surfaces accompanying the model’s dynamics. Cusp catastrophes are a prominent example in catastrophe theory describing hysteresis and possible sudden transitions as a consequence of slightly varying control parameters with applications in population dynamics, mechanical and biological systems^46^,^47^,^48^. The bifurcation lines enclose the hysteresis region and merge at the cusp point. The cusp point is a degenerate critical point where not just the first derivative, but also higher derivatives of the potential function vanish. The degeneracy of this critical point can be unfolded by expanding the potential function as a Taylor series in small perturbations of the parameters *r, p*, and *a* with a characteristic fourth-order polynomial^47^. For a detailed analytical treatment, see Appendices A and B. We define the hysteresis areas enclosed by the bifurcation lines in the parameter range of Fig. 3 (right) as 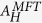 (mean-field for k = 1, *m* = 1) and 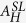 (square lattice). The ratio 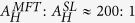 shows that it is significantly less likely for the Euclidean lattice to exhibit the aforementioned hysteresis dynamics in the presence of failure and recovery compared to the mean-field scenario. We will see that this effect can be also observed by comparing a square lattice with a regular random network. We will therefore study the transition from a square lattice to a regular random network by varying the characteristic link length in Sec. *Phase diagrams and transitions for embedded systems*. To obtain the phase diagram we studied the hysteresis behavior and fluctuations for different fixed values of *r* by varying *p*. More details about the Euclidean lattice and its critical behavior for different values of *m* are described in Appendix C. The model’s dynamics is very rich and we show in the section *Oscillatory behavior* that we encounter closed orbits for certain values in the parameter space. We further describe the dynamics and connections to other models, in particular, Schlögl’s first (contact process) and second models and the relation to cusp catastrophes, in Appendix B.

### Spreading dynamics

The dynamics can be driven by the field-like spontaneous failure term or the spread of failure can be triggered by the neighboring failed nodes. We first discuss the dynamics in the hysteresis region of a square lattice with *N* = 50 × 50 nodes, cf. Fig. 2. The two mechanisms are illustrated in video 1 (transition down) and video 2 (transition up). The spontaneous infection term, analogous to an external field, enables the dynamics to form multiple seeds from where the transitions might start. The regions invaded by different seeds expand and move on the lattice. Some of them merge and form larger clusters of active or failed nodes. After some time a stable phase develops.

**Figure 4:**
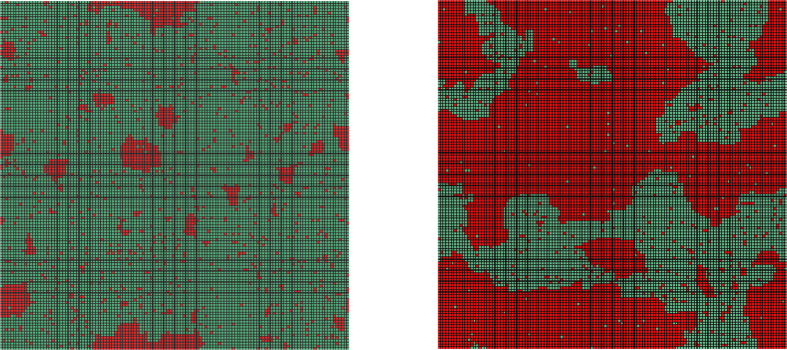
Nucleation for vanishing spontaneous infection. Simulation of the spontaneous recovery model with *p* = 0.05, *q* = 1.0, *r* = 10.0, *q*′ = 0.1 and *m* = 1 on a square lattice with *N* = 128 × 128 nodes. (left) Initially, multiple spreading seeds of failed lattice sites (red) form due to spontaneous failure. (right) Contact dynamics (external failure) dominate and active sites (green) are displaced by failed ones (red). For further details we refer to the video version of the dynamics: video 3 (vanishing spontaneous infection).

In the limit of a vanishing external field we expect nucleation determining the growth of a certain phase. Nucleation is exemplified for a small value of *p* = 0.05 in Fig. 4. The left side of Fig. 4 (left) displays the initially occurring spreading seeds due to the spontaneous infection dynamics. Eventually, contact dynamics (external failure) leads to a local spread of the failure and larger clusters form as illustrated in Fig. 4 (right). A video of the latter example can be found here: video 3 (vanishing spontaneous infection).

### Phase diagrams and transitions for embedded systems

**Figure 5:**
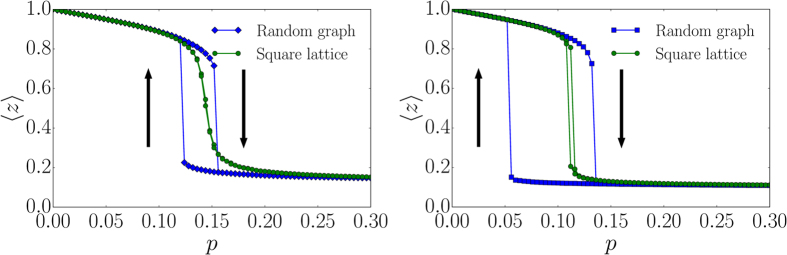
Illustration of hysteresis effects. Simulation of the failure-recovery model (〈*z*〉 average of the fraction of active nodes) with *q* = 1.0, *q*′ = 0.1, *m* = 1 for a square lattice and a regular random graph both with *k* = 4 and *N* = 512 × 512 nodes. (left) The transition is discontinuous for *r* = 0.7 for the random graph but continuous for the square lattice. (right) Only for large *r*/*q*′ (shown for *r* = 1.0) the transition is discontinuous for both the random graph and the square lattice. The black arrows indicate the direction of the simulation loop.

Critical failure-recovery dynamics necessarily occurs close to the hysteresis region. We study the critical transitions for fixed *r* and varying *p* for a regular random graph with degree *k* = 4 and for a square lattice. One observes that for *r* = 0.7 the square lattice shows a continuous transition whereas the random graph exhibits a discontinuous transition (Fig. 5 (left)). Since in real systems control parameters often can be only determined approximately, this demonstrates that critical failure-recovery dynamics on the lattice can be better controlled compared on a random graph. When both paths cross the hysteresis region, e.g., for *r*/*q*′ = 10, both dynamics show a discontinuous transition (Fig. 5 (right)). This however requires parameter tuning, that is, large values of the external spreading rate *r*/*q*′.

**Figure 6:**
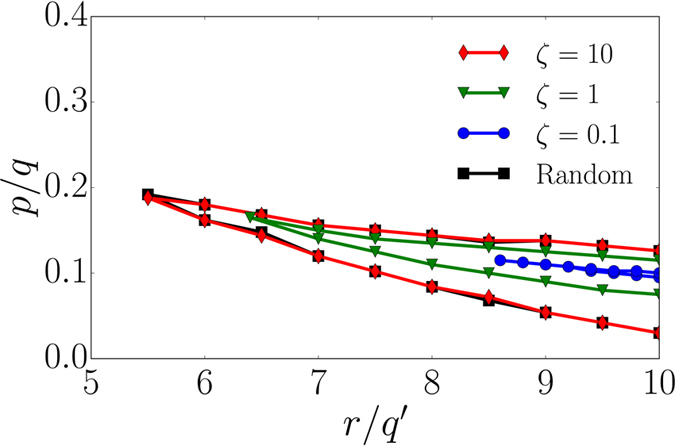
Phase diagrams of spatially embedded networks with *k* = 4 in comparison to a random graph. We set *q* = 1.0, *q*′ = 0.1, *m* = 1 and perform the simulation for networks with degree *k* = 4 and *N* = 250,000 nodes. The phase space of a regular random graph with *k* = 4 (black squares) is compared to an embedded network with the same degree *k* = 4 for different values of the characteristic link length ζ (blue dots, green inverted triangles, red diamonds). For large ζ we obtain the phase space of the regular random graph and for small ζ the square lattice behavior is recovered.

In order to better understand the dramatic differences between random networks and embedded lattices, we analyze here the transition from a square lattice to a regular random graph. To this end, we follow the transition model of Danziger *et al*.^56^ and study the phase space of an embedded system with degree *k* = 4 where randomly chosen nearest-neighbor links are replaced by longer-range links. The lengths *l* of the links are distributed according to an exponentially decaying distribution *P*(*l*) ~ exp(−1/*ζ*), with a link length *l* and characteristic link length *ζ*. In the thermodynamic limit, a square lattice in the limit of *ζ* → 0 is recovered, whereas in the limit of *ζ* → ∞ we obtain a regular random graph (as all link lengths are equally likely). The phase diagrams of an embedded system with *k* = 4 and exponential link length distribution, in the presence of processes (i–iii), are shown in Fig. 6. We clearly observe the transition from a configuration close to a square lattice for *ζ* = 0.1 to that close to a regular random graph for *ζ* = 10. This again illustrates the strong dependence of the extent of the metastable region on the topology. In other words, a variation in the characteristic link length *ζ* causes a very narrow metastable domain (*ζ* = 0.1) to expand into a substantially larger region (*ζ* = 10). Non-local connections induce a faster damage spread in random networks compared to the square lattice where failure cascades are only sustained for a large damage spreading rate *r* within a narrow region of the ratio *p*/*q*. Therefore, the results presented in this section have implications for the understanding of dynamical networks.

### Oscillatory behavior

We will briefly describe the possibility of encountering limit cycles in our dynamics. We investigate this behavior by studying the Lyapunov function^48^ as very recently reported in ref. 57. In our case, the Lyapunov function *V*(*a, u*_*int*_) is derived from the following equations (*α, β* > 0):




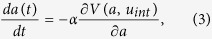







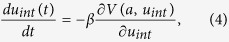




which are equivalent to




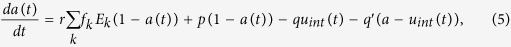







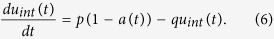




Without loss of generality, we set *β* = 1 and compute *V*(*a, u*_*int*_) to:




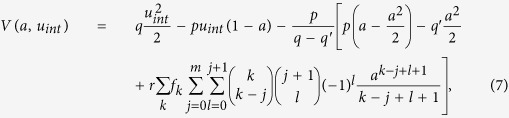




where we used the binomial theorem and set *α* = (*q* − *q*′)/*p*. We find that




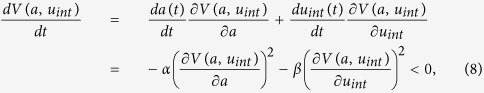




if *q* > *q*′ since *β* = 1.

**Figure 7:**
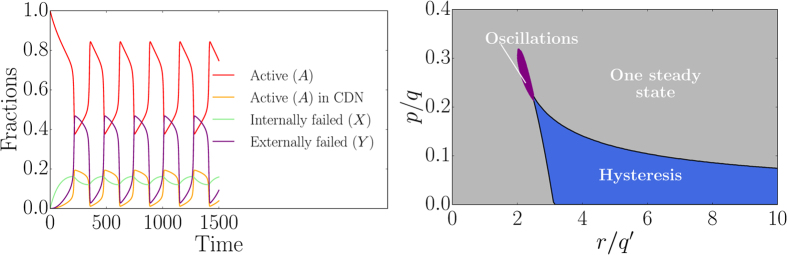
Oscillatory behavior (limit cycles) for *q*′ > q. (left) We show the mean-field time evolution of different compartments, cf. Fig. 1, for *p*/*q* = 19/81, *q* = 0.01, *r*/*q*′ = 3125/1296, *q*′ = 1.0, *k* = 4 and *m* = 1. The values of *p* and *r* correspond to the ones of the bifurcation point as described in Appendix C. We clearly see the oscillatory behavior as a consequence of *q*′ > *q*. (right) The phase space for *k* = 4, *q* = 0.011, *q*′ = 1.0, *m* = 1 while varying *p* and *r* also displays an oscillatory regime (purple). This oscillatory domain has been determined by analyzing the Fourier transform of *z*(*t*) to find the periodic signals.

For *q* > *q*′ we therefore expect no oscillatory behavior, i.e. no closed orbits. However, for *q*′ > *q*, we show in Fig. 7 the existence of closed orbits. As in the phase diagram for *q* > *q*′ illustrated in Fig. 3, we also find a hysteresis region (blue) in Fig. 7 (right) for *q*′ > *q*, where two, in the thermodynamic limit, stable steady states coexist and a region (grey) where only one stable steady state exists. As in Fig. 3, steady states in the grey domain are characterized by a fraction of failed nodes that reaches a low density, a high density or a value in between. However, for *q*′ > *q*, there also exists a regime (purple) where we identified a periodic orbit by analyzing the Fourier transform of the time evolution of the fraction of active nodes. We also show the oscillatory dynamics of a non-embedded regular network in video 4 (oscillations). As discussed in Appendix B, for *q* = *q*′, the differential equations Eqs (1) and (2) can be decoupled and one obtains a single first-order differential equation which has no periodic solutions^48^.

This demonstrates that the phase diagram is substantially more complex than previously believed. Specifically, limit cycles occur for *q*′/*q* < 1 in a narrow region in the phase diagram. This deterministic behavior is markedly different from the stochastic switching dynamics in the hysteresis region but likewise challenges control. It might be difficult to distinguish stochastic switching from limit cycles in some situations where the fraction of active nodes exhibits rapid decay and growth dynamics. However, in contrast to stochastic switching, periodic oscillations even occur for large system sizes what allows the distinction. In addition, for small systems, one could also study the Fourier transform of *z*(*t*) to analyze if the signal is periodic or not.

## Discussion

We have derived a unifying framework for the interplay between failure, damage spread and recovery in spatially embedded and random networks. The theoretical description links diverse phenomena such as complex contagion and phase-switching due to metastability and the occurrence of cusp catastrophes. The number of failed neighbors necessary to allow external failure to act on a node is a crucial parameter of the system. Our analysis revealed that the phase space is substantially more complex than previously known owing to the coexistence of limit cycles and random phase switching within hysteresis.We analytically demonstrated that the mean-field description of the stochastic model systems is equivalent to cusp catastrophes with two bifurcation lines enclosing a metastable domain where two stable stationary states coexist. Inside this metastable region, large fractions of nodes suddenly fail and recover. Our results show that the transition from a random regular network to an embedded network with a short characteristic link length is characterized by a dramatic shrinking of the metastable domain. This suggests that embedded systems with short characteristic link lengths whose dynamics is captured by processes (i–iii) are substantially more robust against abrupt spontaneous and cascading failures compared to non-embedded systems.

Moreover, we have also shown that our theoretical framework is able to describe essential features of the model’s time evolution and that it captures spontaneous failure as an external field in analogy to magnetic systems. However, based on the connection to contact process dynamics we find that the model does not belong to the Ising universality class as conjectured earlier^21^. The arguments in Appendices A and B show the similarities to the (non-equilibrium) contact process belonging to the directed percolation universality class^58^. In fact, as mentioned by Grassberger^59^, relating this dynamics to the Ising universality class would mean an extension of the universality hypothesis from models with detailed balance to models without it. Metastability observed in the hysteresis region results from two effects. For finite systems, unpredictable random phase switching between two unstable states is observed. In the thermodynamic limit, however, small fluctuations in the initial phase of the systems dynamics determine the stationary (stable) state of the system.

Our framework helps to better understand controllability in spatially embedded and random systems where spontaneous recovery, spontaneous failure and cascading failure lead to a remarkably complex dynamic interplay.

## Additional Information

**How to cite this article:** Böttcher, L. *et al*. Failure and recovery in dynamical networks. *Sci. Rep.* **7**, 41729; doi: 10.1038/srep41729 (2017).

**Publisher's note:** Springer Nature remains neutral with regard to jurisdictional claims in published maps and institutional affiliations.

## Supplementary Material

Supplemental Material

Supplementary Movie 1

Supplementary Movie 2

Supplementary Movie 3

Supplementary Movie 4
