# Supplementary material for: Failure and recovery in dynamical networks
Source: Sci Rep. 2017 Feb 3;7:41729. doi: 10.1038/srep41729 (PMC5290536; doi:10.1038/srep41729)
Supplement: Supplemental Material [file srep41729-s1.pdf]

Supplemental Material: Failure and recovery in dynamical networks

L. Böttcher,[1,*] M. Luković,[1,†] J. Nagler,[1,‡] S. Havlin,[2,3] and H. J. Herrmann[1,4]

[1]*ETH Zurich, Wolfgang-Pauli-Strasse 27, CH-8093 Zurich, Switzerland*

[2]*Center for Polymer Studies and Department of Physics, Boston University, Boston, Massachusetts 02215, USA*

[3]*Department of Physics, Bar-Ilan University, 52900 Ramat-Gan, Israel*

[4]*Departamento de Física, Universidade Federal do Ceará, 60451-970 Fortaleza, Ceará, Brazil*

[*] lucasb@ethz.ch

[†] lukovicm@ethz.ch

[‡] jnagler@ethz.ch

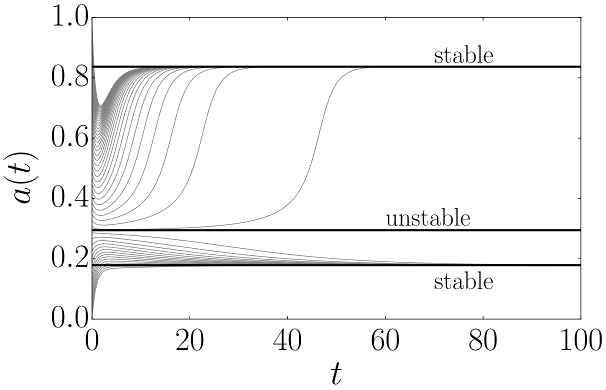

Figure S1. **Convergence to stable states in the hysteresis region.** Mean-field solutions (Euler method) of Eqs. (1) and (2) for $k = 10$, $m = 4$, $r = 0.5$, $p = 0.2$, $q = 1.0$, $q' = 0.1$ and different initial conditions $a(t = 0)$ (grey solid lines). These parameters correspond to a point in the hysteresis region where two stable states and one unstable state (black solid lines) coexist.

Appendix A: Convergence to stable states in the hysteresis region

In the hysteresis region two stable and one unstable states exist. We illustrate the time evolution of trajectories corresponding to different initial conditions in Fig. S1. The grey curves are numerical solutions of the mean-field Eqs. (1) and (2) for $k = 10$, $m = 4$ as applied to approximate the phase-space of a regular random network used in Fig. 3. We clearly see that trajectories which begin above the line of the unstable state converge towards the high-a stable state, $a(t) \to a_{\text{high}}$ for $a(0) > a_{\text{unstable}}$, whereas trajectories starting below the line of the unstable state converge towards the low-a stable state, $a(t) \to a_{\text{high}}$ for $a(0) < a_{\text{unstable}}$.

Appendix B: Connection to other models

To draw a connection to other models we first simplify the two coupled rate equations Eqs. (1) and (2). We therefore set $q = q' = 1$ (excluding limit cycles, cf. Sec. *Oscillatory behavior*) and added Eqs. (1) and (2) to obtain:

$$\frac{da(t)}{dt} = r \sum_k f_k E_k \left(1 - a(t)\right) + p \left(1 - a(t)\right) - a(t). \tag{B1}$$

In particular, we are interested in the stationary states of Eqs. (1) and (2), i.e. $du_{int}/dt = 0$ and $du_{ext}/dt = 0$. If $q \neq q'$, one can still divide the two latter equations describing the stationary states by q and q' to decouple them. This results in rescaled spreading rates

r/q' and p/q instead of r and p as for the case where $q = q' = 1$. For the analysis of the stationary states it is therefore legitimate to set $q = q' = 1$. For $k = 1$, i.e. a network where every node has one temporally changing neighbor, and $m = 0$, we find exact correspondence to the contact process dynamics with spontaneous infection [1]:

$$\frac{da(t)}{dt} = ra(t)\,(1 - a(t)) + p\,(1 - a(t)) - a(t). \tag{B2}$$

The latter equation describes nothing but contact process dynamics with a smeared out second order phase transition transition due to the additional spontaneous infection term. We illustrate the stationary state $a_{st}(r)$ (order parameter) as a function of the external failure rate r in Fig. S2 (left). In the limit of vanishing spontaneous failure $p \to 0$ one encounters a second order phase transition. At the critical point $r_c = 1$ the order parameter grows as $a_{st}(r) \propto (r - r_c)^\beta$ with $\beta = 1$. A non-zero spontaneous failure term leads to a smeared out transition. This situation is similar to the one in the Ising model with an applied magnetic field which also removes the second order phase transition. However, unlike in the Ising model the field equivalent satisfies the condition $p > 0$ and we are restricted to one of the two roots defining the stationary state of Eq. (B2):

$$a_{st}(r, p) = \frac{1}{2r}\left[r - p - 1 + \sqrt{(r - p - 1)^2 + 4rp}\right]. \tag{B3}$$

Close to the critical point $r_c = 1$, i.e. $r \to r_c$, we find $a_{st}(r_c, p) \propto p^{1/\delta_h}$ with the field exponent $\delta_h = 2$ in the mean-field situation.

In order to see the influence of the coupling parameter m on the dynamics, we now turn towards the case $k = 2$ and are free to set $m = 0, 1, 2$. For $m = 2$ all neighborhoods are critically damaged by definition and the stationary state is given by $a_{st}(r, p) = (r + p)/(1 + r + p)$. In particular, this solution is obtained for all regular graphs with degree k and $m = k$ since $E_k = \sum_{j=0}^{k} \binom{k}{k-j} a^{k-j}(1 - a)^j = 1$.

The situation is different for $m = 1$ where at least one neighbor of a given node needs to fail in order to allow external failure acting on the node. This is again in accordance with the contact process where also at least one failed neighbor is necessary to turn on the spreading dynamics. We also find the corresponding exponents $\beta = 1$ and $\delta_h = 2$ in the vicinity of $r_c = 1/2$. In general, we expect this behavior for any regular graph with degree k and $m = k - 1$ since $\lim_{a \to 0} E_k = \sum_{j=0}^{k-1} \binom{k}{k-j} a^{k-j}(1 - a)^j = ka + \mathcal{O}(a^2)$ (at the critical point). That is the reason why we again find the contact process exponents in the latter

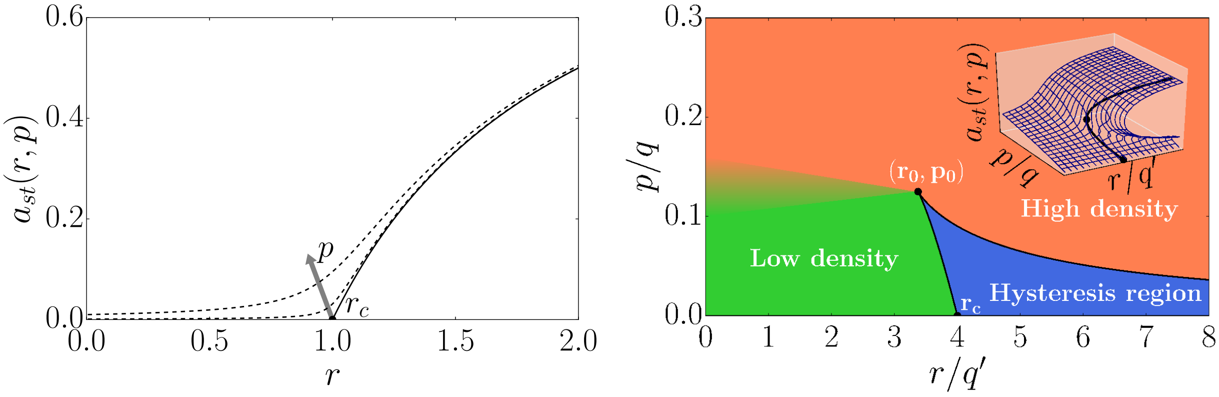

Figure S2. **Analogy to the contact process and Schlögl's second model.** (left) The order parameter $a_{st}(r)$ as a function of the external failure rate r for $k = 1$ and $m = 0$ (mean-field). Analogous to the contact process the black solid line corresponds to the situation where the vanishing spontaneous infection term $p \to 0$ leads to a second-order phase transition at $r = r_c$. The dashed lines show a smeared out transition due to the non-zero spontaneous infection rates $p = 0.001, 0.01$ [1]. (right) The phase space for $k = 2$ and $m = 0$ (mean-field). One clearly sees the hysteresis region where two states coexist (low density and high density failure phases). The spinodals (black solid lines) merge at the bifurcation point $(r_0, p_0) = (27/8, 1/8)$. The critical point, $r_c = 4$ indicates the transition point without additional field-like term ($p = 0$). This situation is similar to Schlögl's second model [2], the phase space of cusp catastrophes or imperfect bifurcations [3].

example and a critical value of $r_c = 1/2$ which is just the critical point of Eq. (B2) divided by k.

Another interesting behavior is found for $m = 0$. Without spontaneous failure term, the rate equation describes a pair-creation contact process [4] and taking this term into account yields a variant of Schlögl's second model [2, 5]. Setting $p = 0$, the stationary state for $r > r_c = 4$ is given by $a_{st}(r) = 1/2(1 + \sqrt{1 - 4/r})$ and $a_{st}(r) = 0$ for $r < r_c$. The phase diagram for $m = 0$ and $p \geq 0$ is illustrated in Fig. S2 (right). Two spinodals define the hysteresis region where two states coexist. As for cusp catastrophes [3], this region is the projection of the hysteresis set from three dimensions into plane space, cf. inset in Fig. S2 (right). In this example, the spinodals are defined by $\Delta = 0$ where the discriminant $\Delta = -r(4 + 4p^3 - r + 4p^2(3 + 2r) + 4p(3 - 5r + r^2))$. For $\Delta < 0$ two stable coexisting steady states exist while for $\Delta > 0$ there is only one. The spinodals merge at the bifurcation point (cusp point) characterized by $(r_0, p_0) = (27/8, 1/8)$ where $\lim_{(r,p)\to(r_0,p_0)} \partial r/\partial a = 0$.

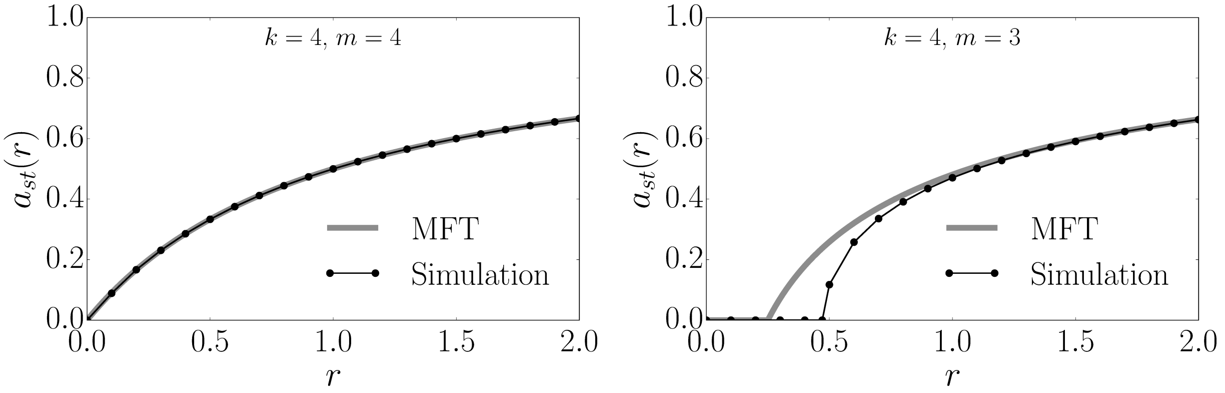

Figure S3. **Growth of the order parameter on a square lattice for different m.** Simulation of the spontaneous recovery model without internal failure dynamics ($p = 0$) and $q' = 1.0$ for different m. (left) The order parameter $a_{st}(r)$ as function of r for $m = 4$ and (right) for $m = 3$. The simulations have been performed on a square lattice with $N = 1024 \times 1024$ nodes.

At the bifurcation point the quantity $\Delta a_{st}(r, p_0) = a_{st}(r, p_0) - a_{st}(r_0, p_0)$ increases with r as $\Delta a_{st}(r, p_0) \propto (r - r_0)^{\tilde{\beta}}$ and with p as $\Delta a_{st}(r_0, p) = a_{st}(r_0, p) - a_{st}(r_0, p_0) \propto (p - p_0)^{1/\tilde{\delta_h}}$ where $a_{st}(r_0, p_0) = 1/3$, $\tilde{\beta} = 1/3$ and $\tilde{\delta_h} = 3$. For this example, it is straightforward to show that the quadratic term in the Taylor expansion of $f(a_{st}, r, p) = r a_{st}^2(1 - a_{st}) + p(1 - a_{st}) - a_{st}$ around $a_{st}(r_0, p_0) = 1/3$ vanishes yielding the characteristic polynomial of the cusp catastrophe [6]. More specifically, the potential of the cusp catastrophe is typically denoted by $V = x^4 + ax^2 + bx$ [6]. The plot of $dV/dx = 0$ shows the typical cusp bifurcation structure. We refer to $dV/dx = 0$ as $f(a_{st}, r, p) = 0$. An expansion of $f(a_{st}, r, p)$ around $(r_0, p_0) = (27/8, 1/8)$ and $a_{st}(r_0, p_0) = 1/3$, i.e. plugging in $r = r_0 + \tilde{r}$, $p = p_0 + \tilde{p}$ and $a_{st} = a_{st}(r_0, p_0) + \tilde{a}_{st}$ yields a third degree polynomial (in this case it is even exact since higher order terms do not exist). For other values of k and m the position of the bifurcation lines will be different. In general, the spontaneous recovery model resembles the dynamics of a modified contact process where a certain minimum number of nodes is necessary to turn on the spreading dynamics [4]. As already mentioned in previous studies and as discussed in the latter examples, slight modifications of the standard contact process dynamics might have dramatic effects on the system's dynamics leading to uncontrollable abrupt transitions [7, 8].

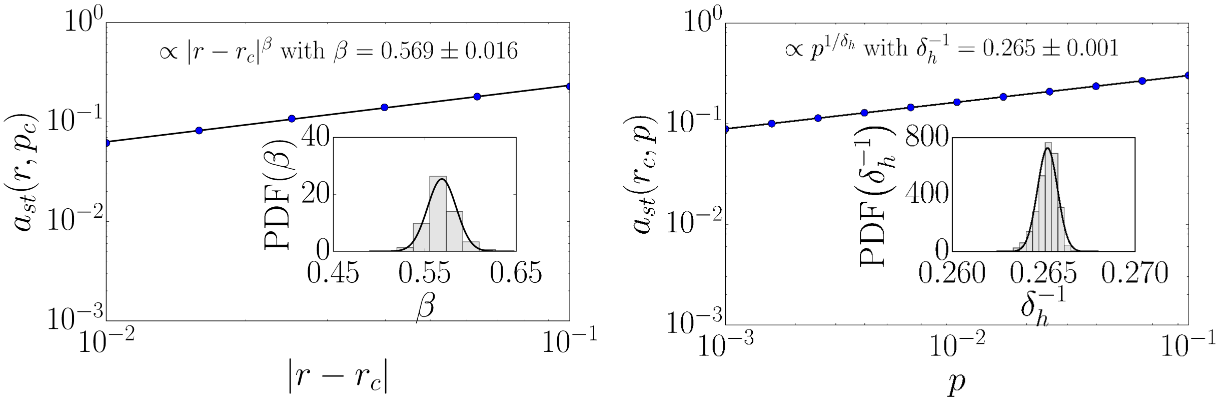

Figure S4. **Critical exponents of the square lattice with $m = 3$.** Simulation of the spontaneous recovery model with $p = 0$, $q' = 1.0$ and $m = 3$. (left) The order parameter a_{st} in the vicinity of the critical point $r_c = 0.47(1)$ for different r. The exponent found indicates contact process dynamics where $\beta = 0.586(14)$ [9]. (right) The order parameter a_{st} at the critical point $r_c = 0.47(1)$ for small values of p. The critical exponent measured also indicates contact process dynamics with $\delta_h^{-1} = 0.285(35)$ [10]. The simulations have been performed on a square lattice with $N = 1024 \times 1024$ nodes (1500 samples). The insets show the PDF's of the exponent's bootstrap analysis.

Appendix C: Critical behavior on the square lattice

We will now study the critical behavior of the dynamics in a system with degree $k = 4$ since there are four nearest-neighbors for every node on a square lattice. Consequently, we have five possibilities of choosing $m = 0, 1, 2, 3, 4$.

We start with the case $m = 4$ for which a CDN even exists when there is no failed neighboring lattice site, i.e. external failure acts all the time independent of the nearest-neighbors' state, cf. Appendix B. Setting $q' = 1.0$, the MFT yields for the stationary state of failed nodes $a_{st}(r) = r/(1 + r)$ (without field-like spontaneous failure). As long as $r > 0$ we find a non-zero fraction of failed nodes in the network. We see in Fig. S3 (left) that the results obtained through simulations on a square lattice are well described by the MFT. An additional field-like contribution of the spontaneous failure p and $q = 1.0$ yields $a_{st}(r) = (r + p)/(1 + r + p)$, cf. Appendix B.

For $m = 3$ we expect to find dynamics analogous to the contact process, since only one failed neighbor is needed to let the neighboring nodes fail. This has been described

in Appendix B and a non-zero stationary state $a_{st}(r)$ is found if $r > r_c$ ($r_c = 1/4$ MFT). From MFT we also find $a_{st}(r) \propto (r - r_c)^\beta$ with $\beta = 1$. At r_c the order parameter grows continuously. Applying the field term in this example one finds $a_{st}(r_c, p) \propto p^{1/\delta_h}$ with $\delta_h = 2$. We show the order parameter $a_{st}(r)$ as a function of r for the square lattice in comparison with MFT in Fig. S3 (right). We also analyzed the critical behavior in the vicinity of the critical point $r_c = 0.47(1)$ of the square lattice (see Fig. S3 (right)). The growth of the order parameter with $\beta = 0.569(16)$ (Fig. S4 (left)) and $\delta_h^{-1} = 0.265(1)$ (Fig. S4 (right)) agrees with the corresponding contact process values $\beta = 0.586(14)$ [9] and $\delta_h^{-1} = 0.285(35)$ [10]. We thus conclude that the model resembles standard contact process dynamics in this case.

For $m = 2$ the transition in MFT is characterized by a jump at $r_c = 1.226$ from zero to $a_{st}(r_c) = 0.322$. In the simulations on the square lattice we found strong dependence on the initial conditions.

We did not find a non-zero value of $a_{st}(r)$ for $m < 2$ having a circle-shaped seed as initial condition on the square lattice. The situations where $m = 0$ or 1 mean that three or four failed neighbors are needed to turn on external failure. Starting from a circle-shaped seed the dynamics will never reach a stable configuration besides the absorbing state (all nodes are active). Nevertheless, we are able to study the dynamics for $m = 1$ as before by introducing the field-like spontaneous failure term again ($p > 0$). In the mean-field situation Eq. (B1) yields for the bifurcation point $(r_0, p_0) = (3125/1296, 19/81) \approx (2.41, 0.23)$ and $a_{st}(r_0, p_0) = 0.4$. This point is also shown in the inset in Fig. 3 (right) of the main article. Similar to the arguments in Appendix B, it is again straightforward to show that the quadratic term in the Taylor expansion of the polynomial describing the stationary states around $a_{st}(r_0, p_0) = 0.4$ vanishes yielding the characteristic polynomial of the cusp catastrophe [6]. The black lines in the latter figure characterize the hysteresis region with two coexisting stationary states similar to Fig. S2 (right). From MFT we find $\Delta a_{st}(r, p_0) \propto |r - r_0|^{\tilde{\beta}}$ with $\tilde{\beta} = 1/3$ and $\Delta a_{st}(r_0, p) \propto |p - p_0|^{1/\tilde{\delta_h}}$ with $\tilde{\delta_h} = 3$. In the square lattice we search for the bifurcation point by first analyzing the hysteresis behavior of the dynamics as shown in Fig. S5 (left). The region where the area defining the multiple states in the hysteresis curve becomes negligible characterizes the vicinity of the cusp point. We then search for the critical point by measuring the fluctuations in that region:

$$\chi_L(r, p) = L^2 \left[\langle a_{st}^2 \rangle - \langle a_{st} \rangle^2 \right] . \quad \text{(C1)}$$

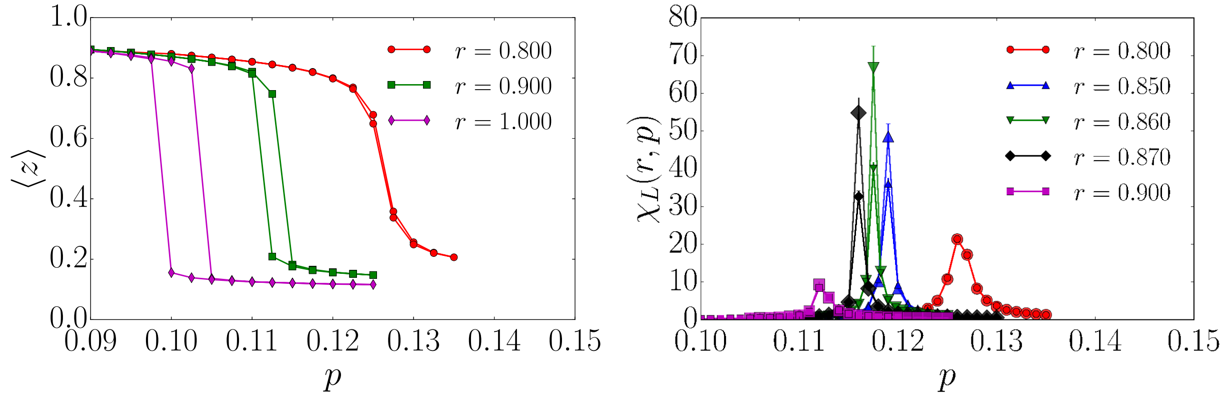

Figure S 5. **Coexisting states and fluctuations on a square lattice for $m = 1$.** (left) Measuring the region of coexisting states by running through a hysteresis-like loop on a square lattice with $N = 2048 \times 2048$ nodes for fixed r and varying p. (right) Fluctuations of the spontaneous recovery model for different fixed values of r and varying p. At around $r = 0.86(1)$ and $p = 0.117(3)$ we find the largest fluctuations corresponding to the bifurcation point. Simulations were performed for $N = 128 \times 128$ (smaller symbols) and $N = 256 \times 256$ (larger symbols) nodes (50 samples).

The fluctuations in the vicinity of the bifurcation point are shown in Fig. S5. We conclude that the cusp point where both spinodals meet is located around $(r_c, p_c) = (0.86(1), 0.117(3))$.

In summary, the arguments in Appendix B and above for the case $m = 3$ (analytical and numerical) show the similarities between our model and the (non-equilibrium) contact process belonging to the directed percolation universality class [11]. However, the exponents β and δ corresponding to the standard contact process are only meaningful for the case where one failed neighbor is sufficient to induce failure. In the case of a more general situation, when more failed neighbors are necessary to sustain failure spread, we are not dealing with the standard directed percolation or contact process universality class but with a more general contact process dynamics [2, 4]. Thus, we do not expect the dynamics to belong to the Ising universality class as conjectured in Ref. [12]. As already mentioned in Ref. [2], if this contact process dynamics belonged to the Ising universality class, it would mean the extension of the universality hypothesis from models with detailed balance to

models without it.

[1] Marro, J. & Dickman, R. *Nonequilibrium Phase Transitions in Lattice Models* (Cambridge University Press, 2005).

[2] Grassberger, P. On Phase Transitions in Schlögl's Second Model. *Z. Phys. B* **47** (1982).

[3] Strogatz, S. H. *Nonlinear dynamics and chaos: with applications to physics, biology, chemistry, and engineering* (Westview press, 2014).

[4] Tomé, T. & de Oliveira, M. J. *Stochastic Dynamics and Irreversibility* (Springer, 2015).

[5] Vellela, M. & Qian, H. Stochastic dynamics and non-equilibrium thermodynamics of a bistable chemical system: the Schlögl model revisited. *J. R. Soc. Interface* **6**, 925–940 (2009).

[6] Zeeman, E. C. *Catastrophe theory* (Springer, 1979).

[7] Böttcher, L., Woolley-Meza, O., Araújo, N. A. M., Herrmann, H. J. & Helbing, D. Disease-induced resource constraints can trigger explosive epidemics. *Sci. Rep.* **5**, 16571 (2015).

[8] Böttcher, L., Woolley-Meza, O., Goles, E., Helbing, D. & Herrmann, H. J. Connectivity disruption sparks explosive epidemic spreading. *Phys. Rev. E* **93**, 042315 (2016).

[9] Moreira, A. G. & Dickman, R. Critical dynamics of the contact process with quenched disorder. *Phys. Rev. E* **54**, R3090–R3093 (1996).

[10] Adler, J. & Duarte, J. A. M. S. Directed percolation: "field" exponents and a test of scaling in two and three dimensions. *Phys. Rev. B* **35**, 7046–7052 (1987).

[11] Henkel, M., Hinrichsen, H. & Lübeck, S. *Non-Equilibrium Phase Transitions Volume I: Absorbing Phase Transitions* (Springer, 2008).

[12] Majdandzic, A. *et al.* Spontaneous recovery in dynamical networks. *Nat. Phys.* **10**, 34–38 (2014).
